# Acute coronary syndrome and acute kidney injury: role of inflammation in worsening renal function

**DOI:** 10.1186/s12872-017-0640-0

**Published:** 2017-07-26

**Authors:** Jorge Ortega-Hernández, Rashidi Springall, Fausto Sánchez-Muñoz, Julio-C. Arana-Martinez, Héctor González-Pacheco, Rafael Bojalil

**Affiliations:** 1 Department of Immunology, Instituto Nacional de Cardiología Ignacio Chávez, Juan Badiano 1, Sección XVI, Tlalpan, 14080 Mexico City, Mexico; 2 Faculty of Medicine, Universidad Nacional Autónoma de Mexico, Avenida Universidad 3000, Copilco-Universidad, 04510 Mexico City, Mexico; 3 Department of Health Care, Universidad Autónoma Metropolitana Xochimilco, Calzada del Hueso 1100, Villa Quietud, Coyoacán, 04960 Mexico City, Mexico; 4 Coronary Care Unit, Instituto Nacional de Cardiología Ignacio Chávez, Tlalpan, 14080 Mexico City, Mexico

**Keywords:** Acute coronary syndrome, Acute renal injury, Cardiorenal crosstalk, Inflammation, Cytokines, Lipid mediators

## Abstract

**Background:**

Acute Kidney Injury (AKI), a common complication of acute coronary syndromes (ACS), is associated with higher mortality and longer hospital stays. The role of cytokines and other mediators is unknown in AKI induced by an ACS (ACS-AKI), leading to several unanswered questions. The worsening of renal function is usually seen as a dichotomous phenomenon instead of a dynamic change, so evaluating changes of the renal function in time may provide valuable information in the ACS-AKI setting. The aim of this study was to explore inflammatory factors associated to de novo kidney injury induced by de novo cardiac injury secondary to ACS.

**Methods:**

One hundred four consecutive patients with ACS were initially included on the time of admission to the Coronary Unit of the Instituto Nacional de Cardiología in Mexico City, from February to May 2016, before any invasive procedure, imaging study, diuretic or anti-platelet therapy. White blood count, hemoglobin, NT-ProBNP, troponin I, C-reactive protein, albumin, glucose, Na^+^, K^+^, blood urea nitrogen (BUN), total cholesterol, HDL, LDL, triglycerides, creatinine (Cr), endothelin-1 (ET-1), leukotriene-B4, matrix metalloproteinase-2 and -9, tissue inhibitor of metalloproteinases-1, resolvin-D1 (RvD1), lipoxin-A4 (LXA4), interleukin-1β, −6, −8, and −10 were measured. We finally enrolled 78 patients, and subsequently we identified 15 patients with ACS-AKI. Correlations were obtained by a Spearman rank test. Low-rank regression, splines regressions, and also protein–protein/chemical interactions and pathways analyses networks were performed.

**Results:**

Positive correlations of ΔCr were found with BUN, admission Cr, GRACE score, IL-1β, IL-6, NT-ProBNP and age, and negative correlations with systolic blood pressure, mean-BP, diastolic-BP and LxA4. In the regression analyses IL-10 and RvD1 had positive non-linear associations with ΔCr. ET-1 had also a positive association. Significant non-linear associations were seen with NT-proBNP, admission Cr, BUN, Na^+^, K^+^, WBC, age, body mass index, GRACE, SBP, mean-BP and Hb.

**Conclusion:**

Inflammation and its components play an important role in the worsening of renal function in ACS. IL-10, ET-1, IL-1β, TnI, RvD1 and LxA4 represent mediators that might be associated with ACS-AKI. IL-6, ET-1, NT-ProBNP might represent crossroads for several physiopathological pathways involved in “*de novo* cardiac injury leading to *de novo* kidney injury”.

**Electronic supplementary material:**

The online version of this article (doi:10.1186/s12872-017-0640-0) contains supplementary material, which is available to authorized users.

## Background

Acute Kidney Injury (AKI) is a common complication of acute coronary syndromes, and its ultimate expression is the cardiorenal syndrome (CRS) type 1, defined “as an acute worsening of heart function leading to AKI or dysfunction” that is a complication of “acute heart failure (AHF) and/or acute coronary syndrome (ACS)”, associated with a high risk mortality and longer hospital stay [1–3].

The heart-kidney axis is complex and usually ill-defined [2]. Complex pathways exist regarding cardiorenal crosstalk; these can be broadly grouped in hemodynamic, hormonal, inflammatory and of immune signaling [4]. Still, the interactions and mechanisms are poorly understood to the point that it has been suggested that the cardiac index in heart failure is not the primary driver of renal dysfunction, leaving the door open to alternative pathways to be explored [5].

The role of various inflammatory mediators has not been fully studied in ACS-AKI [6–8]. A clear example is endothelin-1 (ET-1) that despite its pleiotropic role in endothelial and glomerular dynamics [9, 10] has not been studied. There is also a lack of knowledge of the role of lipid mediators regarding the cardio-renal cross talk, although it has been suggested that specialized pro-resolving lipid mediators (SPMs) such as resolvins (Rv) and lipoxins (Lx) confer protection and repair in experimental models of AKI and myocardial infarction, by limiting the fibrosis and halting the inflammation [11, 12].

Hence, the role of cytokines and mediators is unknown in AKI specifically in the context of ACS, leading to several unanswered questions. Furthermore the worsening renal function is usually seen as a dichotomous phenomenon instead of a dynamic change in the target organ, the kidney, and evaluating the declining kidney’s function may provide more valuable information in the ACS-AKI setting [13].

## Methods

### Aim

The aim of this study was to explore inflammatory factors associated to de novo kidney injury in patients with de novo cardiac injury secondary to ACS.

### Study population

One hundred four consecutive patients were initially included at the time of arrival to the coronary unit of the Instituto Nacional de Cardiología Ignacio Chavez, in Mexico City with ST-elevation myocardial infarction (STEMI) or non-ST elevation acute coronary syndrome (NSTE-ACS), ≥18 years of age and <24 h of the initial symptom, from February to May 2016. All patients’ records were thoroughly examined and reviewed until the patient discharge or dismissal. We enrolled 78 patients, and subsequently we identified 15 patients with ACS-AKI. Seventeen healthy volunteers were included as controls for the serum lipid mediators’ analysis (see Additional file 1: Table S1).

STEMI, NSTE-ACS and key data elements and definitions were defined according to the AHA/ACC guidelines [14–16]. AKI was defined based on serum creatinine (Cr) as stated by the KDIGO guidelines [17]. Delta Cr (ΔCr) was obtained by the subtraction of the baseline value to the admission value. Baseline Cr was defined as the lowest Cr during hospitalization, maintained for more than 72 h or until the patient discharge, and that could not be explained by fluid resuscitation, overload or dialysis.

Patients with known pregnancy or in postpartum period, infectious, autoimmune, hepatic, or neoplastic diseases were excluded; as well as patients with current or previous dialysis, transplant, episodes of AHF or chronic heart failure, and previous ACS. Furthermore, patients with a baseline clearance <55 ml/kg/min^−1^ (by 4-variable standardized-MDRD study equations) were also excluded. We eliminated patients that were diagnosed during the study with any of the exclusion criteria, or loss of information occurred at any time of the study.

The study protocol was approved both by the Research Committee and the Ethics in Research Committee of the Instituto Nacional de Cardiología Ignacio Chavez (#16–971). All patients or their legally authorized representatives provided written informed consent. All procedures were conducted based on the Declaration of Helsinki and local regulations.

### Sample obtaining and serum determinations

Samples were collected on the time of admission before any invasive procedure, imaging study, diuretic or anti-platelet therapy. We obtained between 10 and 20 ml of blood. The serum was centrifuged at 3000 rpm for 25 min, recollected in 500 μL vials and stored at −76 °C until the serum readings were performed.

White blood count (WBC), hemoglobin (Hb), NT-Pro B-Type-Natriuretic-Peptide (NT-ProBNP), troponin-I (TnI), high-sensitive C-reactive protein (hs-CRP), albumin, glucose, sodium (Na^+^), potassium (K^+^), blood urea nitrogen (BUN), high density lipoprotein (HDL), low density lipoprotein (LDL), total cholesterol (TChol), triglycerides (TG) and creatinine (Cr) levels were performed by the hospital clinical laboratory.

ET-1, leukotriene-B4 (LTB4), matrix metalloproteinase (MMP)-2, MMP-9 and tissue inhibitor of metalloproteinases (TIMP)-1 (R&D Systems™, Inc. Minneapolis, MN, USA), RvD1 (Cayman Chemical Company, Ann Arbor, MI, USA), LXA4 (Cloud-Clone Corp. Houston, TX, USA) were determined by enzyme-linked immunosorbent assays using commercial kits per instructions provided by the manufacturer.

And for interleukin (IL)-1β, IL-6, IL-8, IL-10, multiplex magnetic bead-based antibody detection kits were used per the manufacturer’s protocols (Multiplex immunoassay ProcartaPlex® Bender, MedSystems GmbH, Campus Vienna Biocenter 2, Vienna, Austria) and read in Luminex®MAGPIX (Luminex Corporation, Austin, TX) with software v4.2 (Luminex-xPonent-MAGPIX®).

### Statistical analysis

Clinical and laboratorial parameters data were expressed as median and interquartile ranges (IQR). The Χ^2^ test was used to compare clinical characteristics. Mann-Whitney-U test for group comparisons and Kruskal-Wallis test for multiple group comparison were performed. Also, both correlation and regression analysis were performed. Correlations were obtained by a Spearman rank test [18]. In the regression models, cytokines, Pro-BNP, CRP and TnI were entered as log-transformed variables to reduce their skew, being ΔCr the outcome variable. We did not perform an analysis based on the presence of AKI since dichotomization can lead to reduced information quality [19]. Low-rank regression splines were specified in the framework of generalized additive models and fitted to penalized likelihood estimation (GAMPL in SAS) to produce flexible nonparametric regressions, and visually asses the relation with the ΔCr changes the degrees of freedom were determined by generalized cross-validation [20, 21]. The regression models were adjusted to age, sex, diabetes, hypertension history and the type of ACS (STEMI or NSTE-ACS). A *p* value of <0.05 was considered significant. The analysis was done in SPSSv22 and SAS-University-Edition©.

### Protein–protein/chemical interaction and pathway analysis networks

Protein–protein interaction networks were obtained from the STRING v10.0 [22] database, protein–chemical interaction STITCH v4.0 [23] and v5.0 [24] (both with a required confidence (score) >0.7). Also, MetaCore™ (Thomson Reuters) public pathways were also fully reviewed for any possible interactions. All interactions were thoroughly reviewed (from the Co-Mentioned in PubMed Abstracts, references or pathways) for averting as far as possible any false positive or negative edges.

## Results

### Baseline and laboratorial characteristics in ACS vs ACS-AKI

The median age in the ACS-AKI group was 73 years. 73.3% were male, 53.3 had diabetes, 73.3% hypertension, 60% smoking history, 66.7% NSTE-ACS and 33.3% had a fatal outcome during the hospitalization. While in ACS the median age was 58 years, 74.6% were male, 42.9 had diabetes, 55.6% hypertension, 65.1% smoking history and 47.6% NSTE-ACS and 3.2% had a fatal outcome. Only age and death were significant between the groups. Moreover, 4 patients in the ACS group developed AKI during their hospitalization, 2 of them had a fatal outcome which added to those from the ACS-AKI group represent the totality of deceases. Systolic blood pressure (SBP) and GRACE were higher in ACS-AKI (*p =* 0.045 and 0.002, respectively). NT-ProBNP, BUN, admission Cr and, and max Cr during hospitalization, also were higher in the ACS-AKI group (Table 1).

**Table 1:** Clinical and laboratorial characteristics

	ACS	ACS-AKI	*p*
n (%)	63 (80.7)	15 (19.3)	
Age	58 (51–67)	73 (63–78)	0.001
Male (%)	47 (74.6)	11 (73.3)	0.919
DM (%)	27 (42.9)	8 (53.3)	0.463
HTN (%)	35 (55.6)	11 (73.3)	0.136
SMK (%)	41 (65.1)	9 (60)	0.712
NSTE-ACS(%)	30 (47.6)	10 (66.7)	0.185
STEMI (%)	33 (52.4)	5 (33.3)	
Death (%)	2 (3.2)	5 (33.3)	<0.001
BMI kg/m^2^	26.67 (24.22–29.4)	26.56 (23.46–27.55)	0.462
Heart rate	75 (65–81)	75 (64–86)	0.849
SBP mmHg	130.0 (116.0–150.0)	120.0 (100.0–130.0)	0.045
DBP mmHg	80.0 (70.0–90.0)	75.0 (70.0–80.0)	0.206
Mean BP mmHg	96.67 (85.67–110.0)	90.67 (80.0–98.0)	0.103
Hb g/L	14.6 (13.7–15.3)	14 (12.7–15.0)	0.165
WBC ×10^9^/L	9.78 (7.88–12.2)	12.8 (6.51–15.23)	0.351
NT-ProBNP pg/mL	1018.0 (217.7–2567.0)	4746.0 (402.1–8519.0)	0.034
TnI ng/mL	9.92 (0.61–81.56)	11.89 (0.19–37.06)	0.596
hs-CRP mg/L	9.75 (3.44–49.9)	13.77 (5.67–169.79)	0.29
Albumin mg/dL	3.83 (3.6–4.02)	3.82 (3.51–4.04)	0.791
Na^+^ mmol/L	140.0 (138.0–142.0)	140.3 (136.7–144)	0.817
K^+^ mmol/L	4.07 (3.9–4.3)	4.38 (3.93–4.94)	0.101
BUN mg/dL	16.36 (13.3–19.9)	27.18 (21.18–38.0)	<0.001
HDL mg/dL	35.8 (30.08–43.02)	39.38 (30.58–49.7)	0.303
LDL mg/dL	96.63 (79.73–121.86)	102.52 (67.36–111.55)	0.368
T. Chol mg/dL	160.36 (135.68–185.98)	154.19 (122.75–171.27)	0.274
TG mg/dL	133.86 (102.72–197.1)	105.7 (82.74–173.01)	0.159
GRACE	130 (96–152)	156 (134–205)	0.002
Admission Cr mg/dL	0.958 (0.75–1.03)	1.346 (1.02–1.8)	<0.001
Baseline Cr mg/dL	0.803 (0.69–0.95)	0.909 (0.682–1.084)	0.482
ΔCr mg/dL	0.057 (0.0–0.135)	0.411 (0.331–0.8)	<0.001
Max Cr mg/dL	0.97 (0.79–1.14)	1.35 (1.03–1.9)	<0.001

Values are presented in medians and IQR ranges

In the mediators’ analyses, only IL-1β was found increased in the ACS-AKI group (*p =* 0.009) (Table 2). Serum lipid mediators were found different between patients and controls (see Additional file 1: Table S1). Although inflammation has been recently associated with the development of atrial fibrillation [25], only one of our patients developed such arrhythmia after the ACS; this patient did not develop AKI.

**Table 2:** Cytokine and lipid mediator analysis

	ACS	ACS + AKI	*p*
LTB4 pg/mL	1630.04 (1114.72–1968.61)	1711.674 (1092.63–1998.27)	0.751
RvD1 ng/mL	79.02 (78.38–79.87)	79.68 (78.38–80.6)	0.133
LxA4 pg/mL	8.1 (5.91–9.0)	7.23 (4.18–8.59)	0.167
ET-1 pg/mL	59.82 (40.27–80.36)	70.58 (33.43–121.42)	0.435
MMP-2 pg/mL	2497.28 (2033.53–3100.16)	2056.72 (1871.22–2705.97)	0.068
MMP-9 pg/mL	4615.7 (3103.64–5179.12)	5375.66 (3737.82–6030.8)	0.107
TIMP-1 pg/mL	548.93 (420.28–749.69)	544.25 (393.57–647.08)	0.428
IL-1β pg/mL	68.13 (0.55–242.91)	400.1 (114.01–836.05)	0.009
IL-6 pg/mL	2399.67 (928.71–11,629.24)	10,225.0 (1478.48–15,698.29)	0.2
IL-8 pg/mL	823.52 (513.57–1356.21)	958.45 (620.41–2292.41)	0.425
IL-10 pg/mL	743.27 (206.63–1390.77)	338.99 (197.90–24,162.37)	0.929

Values are medians and IQR ranges

### Correlations with ΔCr

The ΔCr had significant positive correlation with BUN (rho = 0.675 *p <* 0.001), admission Cr (rho = 0.645 *p <* 0.001), GRACE (rho = 0.430*p <* 0.001), IL-1β (rho = 0.335 *p =* 0.003), IL-6 (rho = 0.288 *p =* 0.011), NT-ProBNP (rho = 0.257 *p =* 0.029) and age (rho = 0.228 *p =* 0.045). Negative correlations were found with SBP (rho = 0.29 *p =* 0.01), mean-BP (rho = −0.283 *p =* 0.012), diastolic BP (rho = −0.267 *p =* 0.018) and LxA4 (rho = −0.225 *p =* 0.048) (For full correlations see Additional file 2: Table S2).

### Generalized additive models in the worsening of renal function

**Fig. 1:**
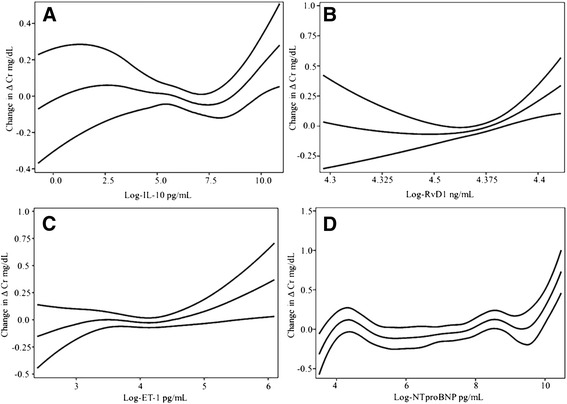
Some of the most noteworthy interactions in the worsening renal function: IL-10 (**a**), RvD1 (**b**), ET-1 (**c**), NT-proBNP (**d**), with CI-95%

**Fig. 2:**
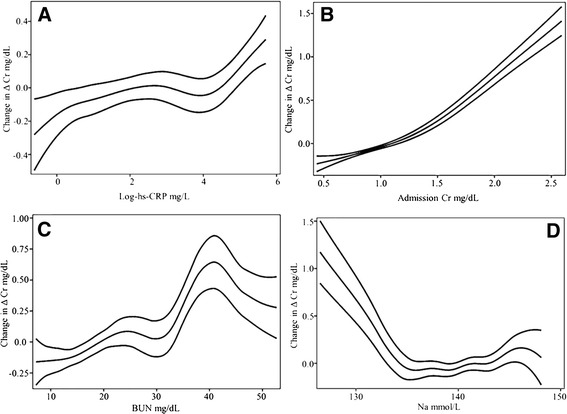
Generalized additive multivariable models in hs-CRP (**a**), admission Cr (**b**), BUN (**c**), Na + (**d**), with CI-95%

IL-10 and RvD1 held outstanding results, both had positive non-linear associations with ΔCr [degree of freedom (*df) =* 3.344 and 2.218 respectively] (For all *df* values please refer to Table 3). In the case of IL-10 a sigmoidal curve was seen, with 2 sharp positive slopes at <1–12 and >1800 pg/mL (logs 0–2.5 and 7.5) (Fig. 1a); regarding RvD1 the steeper positive slope was seen in levels >79.45 ng/mL (log 4.375) (Fig. 1b). ET-1 had also a positive sigmoidal association with ΔCr (*df =* 2.397) (Fig. 1c). NT-proBNP (*df* = 2.953) had 2 sharp positive slopes at the extremes of its concentrations, the first one under 90 pg/mL (log 4.5) and the second one over 2980 pg/mL (log 8) (Fig. 1d). Additionally, hs-CRP had a positive relation to ΔCr, (*df) =* 3.815, and the slope was steeper in values >54.6 mg/L (log 4) (Fig. 2a).

**Table 3:** Generalized additive models

Variable	*Effective df*	*F-value*	P for smoothed term
Age^a^	1.349	5.79	0.005
BMI	4.778	17.79	<0.001
Heart rate	1	0.89	0.348
SBP	3.129	8.52	<0.001
DBP	1	3.87	0.06
Mean BP	4.43	11.95	<0.001
Hb	2.616	13.76	<0.001
WBC	4.05	16.95	<0.001
Admission Cr	3.096	420.36	<0.001
NT-proBNP	2.953	23.37	<0.001
TnI	3.092	13.35	<0.001
CRP	3.815	17.81	<0.001
Alb	1	1.22	0.274
Na	5.501	51.95	<0.001
K	3.244	7	<0.001
BUN	6.031	48.61	<0.001
GRACE	2.496	37.57	<0.001
LTB4	1	0.67	0.418
RvD1	2.218	6.8	<0.001
LxA4	1.878	2.4	0.076
ET1	2.397	6.73	<0.001
MMP2	1	0.1	0.754
MMP9	1	2.68	0.106
TIMP1	1	0	0.963
IL1b	1	1.91	0.171
IL6	1	1.24	0.269
IL8	1	0.86	0.357
IL10	3.344	9.44	<0.001

*Df* Degrees of freedom

Model ΔCr = Predictor + HTN + DM + Type of ACS + Sex + Age

^a^Only included in a partial adjusted analysis (Model ΔCr = Predictor + HTN + DM + Type of ACS + Sex)

**Fig. 3:**
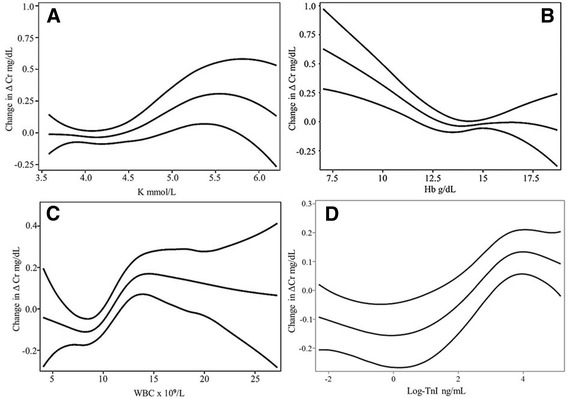
Generalized additive multivariable models in K+ (**a**), Hb (**b**), WBC (**c**), Log-TnI (**d**), with CI-95%

In the case of the renal related parameters, admission Cr (admCr) had an additive log-shaped behavior in the model (*df =* 3.096) (Fig. 2b); BUN had a convoluted regression (*df =* 6.031) but overall it behaved with a positive slope up to 40 mg/dL, being steeper beyond 30 mg/dL, and becoming negative after 40 mg/dL (Fig. 2c). Electrolytes also had significant relations to ΔCr. In the case of Na^+^ the curve, although complex (*df =* 5.501), tended to be L-shaped, with a negative relation below 135 mmol/L (Fig. 2d). K^+^ had a global positive relation (*df =* 3.244), greater beyond 4.5 mmol/L, however, a slight negative slope was seen in concentrations greater than 5.5 mmol/L levels (Fig. 3a). In the case of Hb it had a negative relation to ΔCr (*df* = 2.616) mainly in levels under 12.5 g/dL (Fig. 3b), and white blood count (WBC) in general had a sigmoidal non-linear association (*df* = 4.05), with the positive slope between 10 and 15 × 10^9^/L (Fig. 3c). TnI had also a sigmoidal significant non-linear association (*df* = 3.092) which describes a steep positive slope in values >1 ng/mL (log 0) (Fig. 3d).

**Fig. 4:**
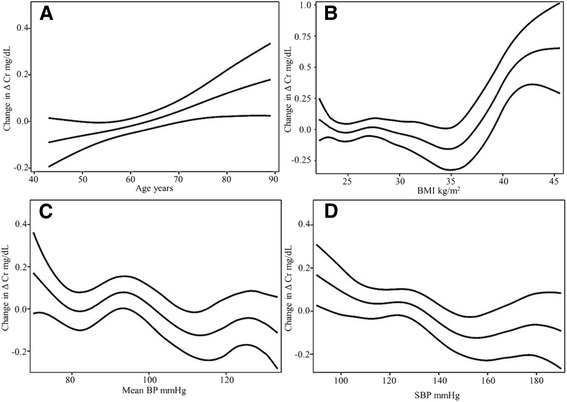
Generalized additive multivariable models in age (**a**), BMI (**b**), Mean BP (**c**), SBP (**d**), with CI-95%

**Fig. 5:**
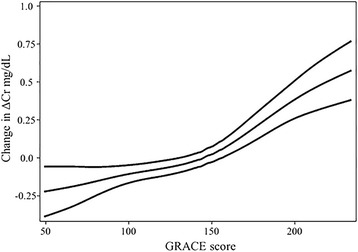
Generalized additive multivariable models in GRACE, with CI-95%

Age had a positive nearly linear relation (*df =* 1.349) to ΔCr (Fig. 4a). In addition, BMI had also a complex significant relationship (*df =* 4.778), and in patients with >35 kg/m^2^ a sharp positive curve was seen (Fig. 4b). Mean (Fig. 4c) and SBP (Fig. 4d) both had a stair-shaped relation (*df =* 4.43 and 3.129) and despite the complexity of their behavior, had an overall negative association to ΔCr. GRACE score likewise had a non-linear positive relationship to ΔCr (*df =* 2.496), especially noticeable in scores >150 points (Fig. 5).

### Protein–protein/chemical interaction and pathways networks analysis

**Fig. 6:**
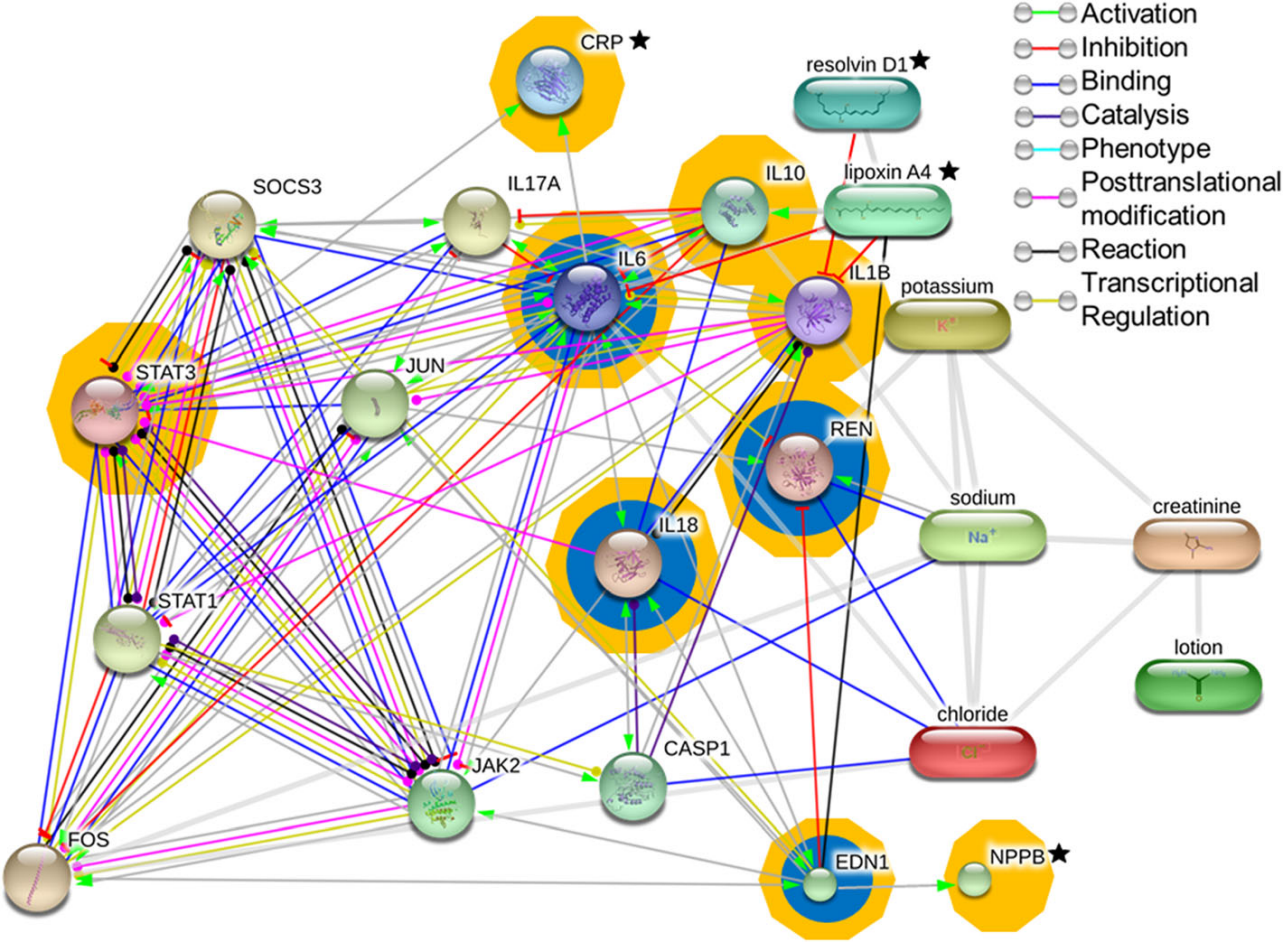
Protein–protein/chemical interaction and pathways networks analysis

Protein and chemical interactions are common in vivo; therefore, we performed a network analysis of the significant variables obtained in this study [IL-1β, IL-6, IL-10, NT-ProBNP (*nppb*), ET-1 (*edn-1*), CRP, LxA4, RvD1, Na^+^, K^+^, Cr, BUN (*lotion*)]. We propose a pathway of predicted protein-protein interactions, based on text-mining, experiments, databases, co-expression, neighborhood, gene fusion, co-occurrence and predictions (STITCHv4.0). Using the STITCHv4.0 database we added 10 protein interactors for the final network, these were obtained by initially adding 20 interactors and further removing receptors and chemical components, leaving only non-receptor proteins (all 10 of them had a score > 0.99). The network analysis showed that the current input data had a strong association with the suppressor of cytokine signaling (SOCS)-3, signal transducer and activator of transcription (STAT)-3, STAT-1, JUN, janus kinase (JAK)-2, FOS, IL-18, IL-17A, renin (REN) and caspase (CASP)-1. Furthermore, we used the Enrichment option included in Chemical Disease Interference System [26] (disease analysis) using “acute myocardial infarction” (orange shade) and “acute renal failure” (blue shade) as the search terms. The final proteins in the network were mapped in STITCHv5.0. We added the following edges that weren’t available in the STITCHv5.0 network: LxA4-IL1b, LxA-IL6, LxA4-IL10, RvD1-IL1b, and IL6-CRP interactions from STITCHv4.0 and EDN1-NPPB from MetaCore™. For the final inclusion in the network, the edges were added only after an extensive review and analysis of the available bibliography in each database; these edges are marked in the node location with a black star (Fig. 6).

## Discussion

In the past, the renal dysfunction induced by cardiac injury was mainly explained through hemodynamic changes. Herein we explored associations with diverse mediators and suggest possible inflammatory and neuro-hormonal pathways in the ACS-AKI context based in our results and on published data. These far from simple pathways that can be traced back to a single triggering insult, are components of complex interaction networks not completely phenotyped yet [4].

### Inflammation and immune signaling pathways

Supporting the notion that an increased inflammation is taking place in the AKI even more so than that seen in ACS alone, IL-1β and IL-6 were associated to ΔCr. As previously reported [27, 28], we found a positive association of IL-6 levels with the worsening of renal function. Previous research in CRS described that higher levels of IL-6 correlate with mortality and kidney injury [29, 30]. Because of its pleiotropic actions, IL-6 might be an important crossroad in the cardio-renal talk, one that might account for the hemodynamic, hormonal and immune pathways of the disease. In ACS-AKI, IL-6 may limit tissue loss by inducing anti-apoptotic pathways; however, the continuous IL-6 expression might also lead to deleterious effects via reduced expression of SOCS3 and increased neutrophilic recruitment [31]. Regarding IL- β it is increased in plasma of patients with AKI [32], and it is produced by endothelial cells and macrophages in coronary arteries in the ischemic heart disease [33]. IL-1β increases leukocyte infiltration and, together with IL-6, stimulates CRP production [34].

Elevated serum levels of IL-10 had also an important positive association with ΔCr. No previous studies have explored the role of IL-10 in the ACS-AKI, but our data agree with previous studies showing that high levels of IL-10 correlate with adverse outcomes in ACS [35] and with the development of AKI after cardiac surgery [36]. Similarly, raised IL-10 levels in renal dysfunction predict mortality in the acute setting [32]. Nonetheless, it has been shown that IL-10 might protect against AKI by inhibiting the inflammatory cytokines and reducing the leukocyte infiltration [37], and in ACS it can reduce heart dysfunction via chronic STAT-3 activation [38]. Thus, high serum levels of IL-10 seem not to be involved directly with the poor outcomes, but could rather represent a counter action to the heightened inflammatory state in ACS-AKI.

A novel finding presented here, is the one seen with SPMs (RvD1 and LxA4). As far as we could acknowledge, no study had previously reported human serum levels of these lipid mediators in ACS or AKI. Both have its biological action by halting the damage and clearing apoptotic neutrophils [11]. In our study, overall low SPMs were seen, and LxA4 exhibited a negative correlation with ΔCr. Notably, in the regression analysis levels of RvD1were found positively associated with ΔCr, however remaining lower than in controls (Additional file 1: Table S1). Recent information from murine models suggests that atherosclerosis is a process with low levels of SPMs, and exogenous administration of RvDs promote the resolution of atherosclerotic lesions [39]. In an ischemia reperfusion model in kidney, SPMs suppress fibrosis [11].

Hs-CRP and WBC are no foreigners to the cardio-renal interactions, our results agree with previous studies that show that raised levels of hs-CRP are independent predictors of AKI by impairing tubular epithelium cell regeneration and altering macrophage polarization [40]. Meanwhile, higher WBC seen in CRS patients can lead to neutrophil mediated injury and dysfunction [41].

### Hormonal pathway

NT-proBNP and ET-1 are part of the so-called hormonal pathway. In the current study, both had a positive relationship to ΔCr, with the highest values having a steeper slope (Figs. 1d and 2c). ET-1 can increase the production of IL-6; even more it precedes the upregulation of IL-1β and IL-6 in AHF. ET-1 is also associated in ACS with microvascular obstruction and lower myocardial salvage [42–44]. ET-1 also upregulates NT-proBNP, which is associated with hypotension, hyponatremia and venous congestion [45, 46], variables associated with the hemodynamic explanation of CRS-1. TnI was also found associated to the worsening of renal function in ACS; previous studies demonstrated that AKI alone can raise the levels of TnI indicating that AKI can also lead to a cardiac injury and that these effects seem to be bidirectional [47].

### Hemodynamic pathways

In our data Hb had a negative correlation to ΔCr in which a steeper slope was seen in the values <12.5 g/dL. Anemia can be part of a dreadful combination in the so call cardio-renal-anemia syndrome since it carries a poorer prognosis, and its prevalence can reach just over 20% in patients with chronic HF [48]. In addition, GRACE along with some of its elements were significantly associated with ΔCr; in previous studies a GRACE >160 was related to the development of AKI [49].

### Anthropometric factors

BMI had a significant relation with ΔCr in our study, mainly after 30 kg/m^2^. The so called “obesity paradox” has been a point of controversy in ACS [50], but in AKI the paradox might not be so, since this and other studies suggest that an increased BMI is a risk factor for developing AKI [51].

### Study limitations

Our study is not exempt of limitations. Previous studies suggest that sampling timing might influence the measurements [35, 52], and although the sample was obtained at the time of arrival to the emergency department, and all patients were recruited within the first 24 h of the initial symptoms, no further subgroup analysis was performed because the study was not designed for this purpose. However, since time of ischemia has been linked to inflammation and renal function [53], it would be interesting to explore whether or not the fact that patients had had previous symptoms of angina, or larger lag periods for initiating therapy, would influence the possible outcome towards AKI. A weakness in our study is the lack of long term outcome data. Also, the associations presented here, although compelling, do not represent causation. Strong points in our study are the fact that it looks at the dynamic changes of ΔCr in ACS-AKI; and that the samples were collected before any invasive procedure, imaging study, diuretic or anti-platelet therapy, thus, precluding their influence on the results as has been demonstrated [54].

## Conclusions

In conclusion, our data suggest that inflammation and its components play an important role in ACS-AKI and the worsening of renal function in the ACS setting. IL-10, ET-1, IL-1β, TnI, RvD1 and LxA4 represent mediators that might be associated with cardiorenal crosstalk. Furthermore, IL-6, ET-1, NT-ProBNP possibly represent crossroads for several physiopathological pathways involved in “*de novo* cardiac injury leading to *de novo* kidney injury”.

## Additional files

Additional file 1: Table S1. Results containing the contrast in serum lipid mediator’s levels against controls. (DOCX 63 kb)

Additional file 2: Table S2. Spearman’s rank correlations between all studied variables and Δ Cr. (DOCX 79 kb)

## Abbreviations

ACS: Acute coronary syndrome
AHF: Acute heart failure
AKI: Acute Kidney Injury
BP: Blood pressure
BUN: Blood urea nitrogen
CASP: Caspase
Cr: Creatinine
CRS: Cardiorenal syndrome
DBP: Diastolic Blood Pressure
df: Degree of freedom
ET-1: Endothelin-1
Hb: Hemoglobin
HDL: High density lipoprotein
hs-CRP: High-Sensitive C-Reactive-Protein
IL: Interleukin
IQR: Interquartile range
JAK: Janus kinase
K +: Potassium
LDL: Low density lipoprotein
LTB4: Leukotriene-B4
Lx: Lipoxin
MMP: Matrix metalloproteinase
Na +: Sodium
NSTE-ACS: Non-ST elevation acute coronary syndrome
NT-proBNP: NT-ProB-Type-Natriuretic-Peptide
REN: Renin
Rv: Resolvin
SBP: Systolic blood pressure
SOCS: Suppressor of cytokine signaling
SPMs: Specialized pro-resolving lipid mediators
STAT: Signal transducer and activator of transcription
STEMI: ST-elevation myocardial infarction
TChol: Total cholesterol
TG: Triglycerides
TIMP: Tissue inhibitor of metalloproteinases
TnI: Troponin-I
WBC: White blood count
ΔCr: Delta Cr
