# Supplementary material for: Acute coronary syndrome and acute kidney injury: role of inflammation in worsening renal function
Source: BMC Cardiovasc Disord. 2017 Jul 26;17:202. doi: 10.1186/s12872-017-0640-0 (PMC5530514; doi:10.1186/s12872-017-0640-0)
Supplement: Supplementary file 1 — Results containing the contrast in serum lipid mediator’s levels against controls. (DOCX 63 kb) [file 12872_2017_640_MOESM1_ESM.docx]

**Additional File 1**

**Table S1:** Serum lipid mediators in patients and controls.

|  | ACS | ACS + AKI | Controls | p |
| --- | --- | --- | --- | --- |
| LTB4 | 1630.04  (1114.72-1968.61) * | 1711.674  (1092.63-1998.27) * | 158.15  (134.65-174.93) | <0.001 |
| RvD1 | 79.02  (78.38-79.87) * | 79.68  (78.38-80.6) * | 85.91  (85.84-85.98) | <0.001 |
| LxA4 | 8.1  (5.91-9.0) * | 7.23  (4.18-8.59) * | 9.17  (9.16-9.19) | <0.001 |

Significant differences were found in SPMs between patients and controls. Serum levels of LTB4 were lower and those of RvD1 and LxA4 were higher in the control group. **p<*0.05 with Dunn’s test for pairwise comparison against controls.
