# Supplementary material for: Acute coronary syndrome and acute kidney injury: role of inflammation in worsening renal function
Source: BMC Cardiovasc Disord. 2017 Jul 26;17:202. doi: 10.1186/s12872-017-0640-0 (PMC5530514; doi:10.1186/s12872-017-0640-0)
Supplement: Supplementary file 2 — Spearman’s rank correlations between all studied variables and Δ Cr. (DOCX 79 kb) [file 12872_2017_640_MOESM2_ESM.docx]

**Additional File 2**

**Table S2:** Spearman’s rank correlations with Δ Cr

| Variable | Spearman | p | Variable | Spearman | p |
| --- | --- | --- | --- | --- | --- |
| Age | 0.228 | 0.045 | **Na** | 0.204 | 0.075 |
| Sex | 0.087 | 0.451 | **K** | 0.22 | 0.053 |
| DM | 0.097 | 0.397 | **GRACE** | 0.43 | <0.001 |
| HTN | 0.155 | 0.176 | **LTB4** | -0.03 | 0.797 |
| SMK | 0.057 | 0.618 | **RvD1** | 0.046 | 0.691 |
| BMI | -0.004 | 0.976 | **LxA4** | -0.225 | 0.048 |
| Heartrate | 0.009 | 0.935 | **ET1** | 0.154 | 0.178 |
| SBP | -0.29 | 0.01 | **MMP2** | -0.015 | 0.899 |
| DBP | -0.267 | 0.018 | **MMP9** | 0.189 | 0.097 |
| Mean BP | -0.283 | 0.012 | **TIMP1** | -0.005 | 0.963 |
| Hb | -0.024 | 0.836 | **IL1b** | 0.335 | 0.003 |
| WBC | 0.192 | 0.092 | **IL6** | 0.288 | 0.011 |
| Cr | 0.645 | <0.001 | **IL8** | 0.115 | 0.318 |
| NT-proBNP | 0.257 | 0.029 | **IL10** | 0.12 | 0.294 |
| TnI | 0.071 | 0.542 | **Alb** | -0.1 | 0.415 |
| CRP | 0.296 | 0.013 |  |  |  |
